# Role of insulin-like growth factor 1, sex and corticosteroid hormones in male major depressive disorder

**DOI:** 10.1186/s12888-021-03116-2

**Published:** 2021-03-17

**Authors:** Hiroshi Arinami, Yutaro Suzuki, Misuzu Tajiri, Nobuto Tsuneyama, Toshiyuki Someya

**Affiliations:** Department of Psychiatry, Niigata University Graduate School of Medical and Dental Sciences, 1-757 Asahimachi-dori, Chuo-ku, Niigata, 951-8510 Japan

**Keywords:** Cortisol, DHEAS, Estradiol, IGF1, Testosterone

## Abstract

**Background:**

Hormones of the hypothalamic–pituitary–gonadal (HPG), hypothalamic–pituitary–adrenal (HPA), and hypothalamic–pituitary–somatotropic (HPS) axes are potentially involved in major depressive disorder (MDD), but these hormones have not been simultaneously investigated in male patients with MDD. We investigated the association between male MDD symptoms and estradiol, testosterone, cortisol, dehydroepiandrosterone sulfate (DHEAS), and insulin-like growth factor 1 (IGF1).

**Methods:**

Serum estradiol, testosterone, cortisol, DHEAS, and IGF1 levels were measured in 54 male patients with MDD and 37 male controls and were compared with clinical factors. We investigated the associations between hormone levels and Hamilton Depression Rating Scale (HAM-D) scores. The correlations among hormones were also investigated.

**Results:**

Patients had significantly lower estradiol levels than controls (22.4 ± 8.4 pg/mL vs. 26.1 ± 8.5 pg/mL, *P* = 0.040). Serum estradiol levels were negatively correlated with HAM-D scores (*P* = 0.000094) and positively correlated with Global Assessment of Functioning scores (*P* = 0.000299). IGF1 levels and the cortisol:DHEAS ratio were higher in patients than in controls (IGF1: 171.5 ± 61.8 ng/mL vs. 144.1 ± 39.2 ng/mL, *P* = 0.011; cortisol:DHEAS ratio: 0.07 ± 0.05 vs. 0.04 ± 0.02, *P* = 0.001). DHEAS levels were lower in patients than in controls (227.9 ± 108.4 μg/dL vs. 307.4 ± 131.2 μg/dL, *P* = 0.002). IGF1, cortisol:DHEAS ratio, and DHEAS were not significantly correlated with HAM-D scores. Cortisol and testosterone levels were not significantly different between patients and controls. Serum estradiol levels were positively correlated with DHEAS levels (*P* = 0.00062) in patients, but were not significantly correlated with DHEAS levels in controls.

**Conclusion:**

Estradiol may affect the pathogenesis and severity of patients with MDD in men, and other hormones, such as those in the HPA and HPS axes, may also be involved in male MDD. Additionally, a correlation between estradiol and DHEAS may affect the pathology of MDD in men.

## Introduction

The incidence of major depressive disorder (MDD) differs by sex [1]; therefore, the pathogenesis of MDD is likely to be affected by sex hormones of the hypothalamic–pituitary–gonadal (HPG) axis. The association between testosterone and MDD in men has been studied [1, 2], and testosterone receptors have been found to be present in numerous brain regions associated with mood, such as the hippocampus and amygdala [1]. In recent years, the association between estradiol and men has also been reported. In studies of men with hypogonadism or gender identity disorder (male to female), estrogen treatment has reduced stress responses and symptoms of depression [3–6]. However, few studies have investigated the relationship between symptom severity in male patients with MDD and estradiol, including testosterone.

The hypothalamus–pituitary–adrenal (HPA) axis has been widely studied with respect to the pathogenesis of MDD. In patients with MDD, increased cortisol and decreased dehydroepiandrosterone sulfate (DHEAS) have been reported [7–11]. Through anti-glucocorticoid effects, DHEAS has protective effects against the neurotoxicity of cortisol [11]; therefore, changes in the cortisol:DHEAS ratio may represent a sensitive indicator of HPA function [12]. More recently, the association between the HPA and HPG axes has been examined [13–15], but few studies have simultaneously considered the HPA and HPG axes in male patients with MDD.

Insulin-like growth factor 1 (IGF1) is produced in the hypothalamic–pituitary–somatotropic (HPS) system and is suggested to be involved in the pathogenesis of MDD through its neuroprotective, neurogenic, and anti-inflammatory effects [16]. Although associations between the HPS and HPG axes have been reported [17, 18], these relationships in male MDD are not well understood.

In addition to sex hormones, the HPA and HPS axes vary between men and women [13, 19, 20], but the relationship between MDD and these hormones in men is not well understood because most previous studies have involved women. Moreover, the combined role of each hormone and the relationships among hormones in male MDD are unclear because no studies have examined the HPA, HPS, or HPG axes simultaneously. The aim of this study, therefore, was to clarify the role of hormones in male MDD and the relationships between these hormones.

## Methods

### Participants

We studied 54 male patients with MDD attending or hospitalized at the Niigata University Medical & Dental Hospital and 37 healthy male volunteers. All participants were aged 18–65 years. Patients were diagnosed with MDD according to the criteria specified in the *Diagnostic and Statistical Manual of Mental Disorders, Fifth Edition* and were not combined with other psychiatric diagnoses, such as drug or alcohol abuse, bipolar disorder, delusional disorder, anxiety disorder, and dementia. Exclusion criteria included a history of serious physical diseases (e.g., malignancy; infection; autoimmune disease; and heart, lung, liver, gastrointestinal, kidney, neurological, and endocrine disease), and a history of steroid hormone use. The patients received individualized treatment from clinicians.

This study was approved by the Genetics Ethics Committee of Niigata University Graduate School of Medical and Dental Sciences (approval number G - 0758) and followed the ethical principles of the Declaration of Helsinki.

All participants were informed of the study’s aims and procedures and provided written informed consent before participating in the study. Informed consent was obtained from parents and/or legal guardian of patients with MDD if the patient’s capacity to provide consent was compromised.

### Assessment of symptoms in patients with MDD

The mental status of patients was assessed by a psychiatrist who was not the patient’s attending physician. Symptoms of depression were assessed using the Hamilton Depression Rating Scale (HAM-D). Social, occupational, and psychological functioning were assessed using the Global Assessment of Functioning (GAF) scale [21]. Scores of the GAF scale range from 1 to 100, with scores closer to 100 indicating better function.

### Blood samples and physical measurements

All blood samples were collected in the morning after at least 8 h of fasting. Smoking, exercise, and activities that could lead to excessive stress, such as work, were prohibited on the morning before blood samples were collected. Serum samples were centrifuged at 4 °C and stored at − 80 °C. Sera were analyzed using standard methods (SRL Inc., Tokyo, Japan). The body mass index (BMI) of participants was measured when blood samples were collected.

### Data analysis and statistics

Unpaired *t*-tests and chi-squared tests were used to compare the patient group with the control group. Correlations between hormone levels, HAM-D, and GAF were analyzed using the Pearson correlation coefficient. To investigate the factors affecting HAM-D, stepwise multiple regression analysis was performed using serum estradiol level, age, BMI, type of antidepressant, total imipramine equivalence, and duration of illness as independent variables. To investigate the correlation between hormones, multiple regression analysis was performed using estradiol and IGF1 as dependent variables, age, BMI, and each hormone level as independent variables. *P* values of less than 0.05 were considered statistically significant. All analyses were performed using IBM SPSS 25 (IBM Japan, Tokyo, Japan).

## Results

The clinical characteristics of the included patients are shown in Table 1. As shown in Table 2, in terms of age, BMI, smoking history, and glycated hemoglobin (HbA1c), there were no significant differences between the two groups. Serum estradiol levels and serum DHEAS levels were lower in the MDD group than in the control group (Table 2). The cortisol:DHEAS ratio and serum IGF1 levels in the MDD group were higher than those in the control group (Table 2). No significant differences in serum cortisol levels and serum testosterone levels were found between the MDD group and control group (Table 2).

**Table 1:** Clinical characteristics of patients with MDD

Variables		
Age^a^	year	42.4 ± 14.7
Body mass index^a^	kg/m^2^	24.2 ± 3.1
Smoker / non smoker	n	13 / 41
HAM-D^a^		19.3 ± 7.0
GAF^a^		32.4 ± 7.6
Duration of illness^a^	year	6.2 ± 7.9
No. depressive episodes^a^		1.7 ± 1.1
Antidepressant use	n (%)	39 (72.2)
TCA	n (%)	1 (1.8)
SSRI	n (%)	27 (50.0)
SNRI	n (%)	7 (13.0)
NaSSA	n (%)	4 (7.4)
Imipramine equivalence^a^	mg/day	110.7 ± 95.4
Benzodiazepine use	n (%)	17 (31.5)
Severity/course specifier at first medical examinationenzodiazepine use		
Mild	n (%)	14 (25.9)
Moderate	n (%)	29 (53.7)
Severe	n (%)	5 (9.2)
With psychotic features	n (%)	3 (5.6)
In partial remission	n (%)	3 (5.6)

^a^Values are presented as mean ± standard deviation

Abbreviations: *HAM-D* Hamilton Rating Scale for Depression, *TCA* Tricyclic antidepressants, *SSRI* Selective serotonin reuptake inhibitors, *SNRI* Serotonin-norepinephrine reuptake inhibitors, *NaSSA* Noradrenergic and specific serotonergic sntidepressant

**Table 2:** Comparative profile of male patients with MDD and control participants

Variables	MDD^1^	Control	*P* value
Number	54	37	–
Age (year)^a^	42.4 ± 14.7	39.4 ± 7.0	*P* = 0.187†
Body mass index (kg/m^2^)^a^	24.2 ± 3.1	24.0 ± 3.5	*P* = 0.661†
Smoker/non smoker	13 / 41	5 / 32	*P* = 0.214‡
HbA1c (%)^a4^	5.4 ± 0.5	5.4 ± 0.8	*P* = 0.970†
Cortisol (μg/dl)^a^	12.1 ± 4.8	11.1 ± 3.0	*P* = 0.191†
DHEAS (μg/dl)^a2^	227.9 ± 108.4	307.4 ± 131.2	*P* = 0.002†
Cortisol / DHEAS ratio^a^	0.07 ± 0.05	0.04 ± 0.02	*P* = 0.001†
IGF1 (ng/ml)^a3^	171.5 ± 61.8	144.1 ± 39.2	*P* = 0.011†
Testosterone (ng/ml)^a^	5.0 ± 1.6	4.9 ± 1.6	*P* = 0.709†
Estradiol (pg/ml)^a^	22.4 ± 8.4	26.1 ± 8.5	*P* = 0.040†

^a^Data are expressed as the mean ± standard deviation. †Unpaired t-test. ‡Chi-squared tests. Abbreviations: ^1^*MDD* Major depressive disorder, ^2^*DHEAS* Dehydroepiandrosterone and its sulfate, ^3^*IGF1* Insulin-like growth factor 1, ^4^*HbA1c* Glycated hemoglobin

**Fig. 1:**
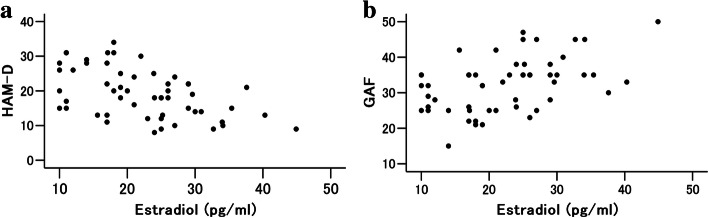
Scatterplot of serum estradiol versus HAM-D (**a**) and serum estradiol versus GAF scores (**b**) in male patients with MDD. Serum estradiol showed significant negative correlation with HAM-D scores (*P* = 0.0000940) (**a**) and significant positive correlation with GAF scores (*P* = 0.000299) (**b**)

On the basis of Pearson correlation analysis, serum estradiol levels were negatively correlated with HAM-D scores (R = − 0.506, *P* < 0.001) (Fig. 1a) and positively correlated with GAF scores (R = 0.473, *P* < 0.001) (Fig. 1b). Serum cortisol levels, serum DHEAS levels, serum IGF1 levels, and serum testosterone levels were not significantly correlated with HAM-D scores. Stepwise multiple regression analysis indicated that as serum estradiol levels decreased, HAM-D scores increased (Table 3).

**Table 3:** Stepwise multiple regression analysis of the relationship between HAM-D scores and dependent variables

Independent Variables	Adjusted *R*^*2*^	*Constant*	*F*	*β*	SE	*P*
Total mode	0.242	28.827	17.916			0.000094
Estradiol				−0.424	0.100	

According to Pearson’s correlation analysis, patients’ estradiol levels were positively correlated with DHEAS levels (R = 0.332, *P* = 0.014) and with testosterone levels (R = 0.423, *P* = 0.001); IGF1 levels were positively correlated with DHEAS levels (R = 0.451, *P* = 0.001). No significant differences were found between other hormones in patients. In controls, estradiol levels were positively correlated with testosterone levels (R = 0.416, *P* = 0.01), but no significant differences were found between other hormones, including estradiol and IGF1 levels. Stepwise multiple regression analysis showed no correlation between IGF1 and DHEAS, whereas estradiol was correlated with DHEAS and testosterone (Table 4).

**Table 4:** Stepwise multiple regression analysis of the relationship between estradiol levels and dependent variables in patients

Independent Variables	Adjusted *R*^*2*^	*Constant*	*F*	*β*	SE	*P*
Total mode	0.289	3.880	11.786			0.000062
Testosterone				2.388	0.610	
DHEAS				0.029	0.009	

## Discussion

### Serum estradiol levels in male patients with MDD

In the current study, serum estradiol levels were negatively correlated with the severity of depression in male patients. In a previous study among healthy men, plasma estradiol levels were positively correlated with serotonin 2A receptor (5-HT 2AR) binding in the cerebral cortex [22], which is consistent with the results of our study. However, no previous study has shown a correlation between estradiol and severity of MDD in males, as in our study.

Several studies have shown that estradiol is involved with the pathogenesis of male MDD, and we also found that serum estradiol levels in male patients were lower than in male controls. Through estradiol supplementation, depression-like behaviors induced by gonadectomy were improved in male rats [23]. In a different study of male mice, activation of estrogen receptor β in the striatum increased serotonin levels and showed antidepressant effects [24]. Furthermore, the administration of estradiol in male rats increased the binding site densities of 5-HT 2AR and serotonin transporter in the dorsal raphe nucleus [25–27]. In a clinical study, Eskelinen et al. [28] compared serum estradiol levels in 74 elderly men with depressive disorders and 367 controls; consistent with our results, serum estradiol levels were lower in depressed patients than in controls. However, several studies of male patients with MDD have found no significant differences in serum estradiol levels between patients and controls [29–31]. It should be noted that all of those studies had small sample sizes, with the maximum including 22 male patients with MDD. Our study included 54 patients, a larger sample size than those past studies, which may be the reason for the difference in results.

### IGF1, DHEAS, cortisol:DHEAS ratio, cortisol, and testosterone in male patients with MDD

The patients in the current study had higher serum IGF1 levels and lower serum DHEAS levels than the controls. The cortisol:DHEAS ratio was higher in patients, but serum cortisol and testosterone levels were not different.

In a meta-analysis for patients with MDD and bipolar disorder [16], as well as a meta-analysis of patients with MDD [11], serum IGF1 levels were higher and serum DHEAS levels were lower in patients than in controls. Therefore, our study supports these previous results analyzing both men and women, and we hypothesize that there is an association between estradiol, IGF1, and DHEAS in male MDD. Several non-MDD studies have shown an association of these hormones with other conditions. A study of men with congenital aromatase deficiency indicated that estradiol regulates the production of IGF1 [18]. In a study including 12,300 men in the general population, estradiol was positively correlated with DHEAS [15]. Therefore, it is possible that estradiol is co-regulated with IGF1 and DHEAS. Furthermore, our study showed a positive correlation between estradiol and DHEAS in male patients with MDD but not in controls. To determine whether the relationships between these hormones, or differences in the regulatory function of MDD and controls, are related to the pathology of male MDD, further studies are needed.

Activation of the HPA axis is widely recognized as one of the factors involved in the pathogenesis of MDD [7–10, 12], and an increased cortisol:DHEAS ratio in MDD has been demonstrated [12]. Our study also showed an increase in the cortisol:DHEAS ratio, although no increase was observed in serum cortisol levels. HPA axis function can also be decreased in depressed patients [32, 33]. As described above, results linking the HPA axis with MDD are inconsistent. However, some studies have shown that the HPA axis function may be reduced using antidepressants [8, 34, 35] and that this may be different for melancholic and atypical depression [36]. Our study allowed the use of antidepressants and did not distinguish between the subtypes of MDD; therefore, further research is needed to consider these factors.

Regarding testosterone, several studies have shown a relationship between a decrease in testosterone and depression, but the results are inconsistent [2, 37–39]. In a recent study using male rats, antidepressant-like effects of testosterone were shown to be partly mediated by its conversion to estradiol [23]. As our study demonstrated the relationship between estradiol and testosterone, further analysis involving estradiol and testosterone in male MDD is warranted.

### Limitations of this study

The current study had several limitations. First, our sample size was small, so detectability may have been limited. Additionally, the use of antidepressant medication may have been a confounding factor. Second, we did not classify the subtypes of depression. Third, we did not take into account psychosocial factors (e.g., employment status), which could be confounding factors for cortisol levels.

## Conclusion

Estradiol may affect the pathogenesis and severity of MDD in men and other hormones, such as those in the HPA and HPS axes, may also be involved in male MDD. Furthermore, the correlation between estradiol and DHEAS may affect the pathology of MDD in men. Further research is needed to examine the relationship between estradiol and these other hormones in male MDD.

## Data Availability

All data generated or analyzed during this study are included in this published article.

## Abbreviations

BMI: Body mass index
DHEAS: Dehydroepiandrosterone sulfate
GAF: Global Assessment of Functioning
HAM-D: Hamilton Depression Rating Scale
HPA: Hypothalamic–pituitary–adrenal
HPG: Hormones of the hypothalamic–pituitary–gonadal
HPS: Hypothalamic–pituitary–somatotropic
IGF1: Insulin-like growth factor 1
MDD: Major depressive disorder
